# Integrin α5 subunit is required for the tumor supportive role of fibroblasts in colorectal adenocarcinoma and serves as a potential stroma prognostic marker

**DOI:** 10.1002/1878-0261.12583

**Published:** 2019-11-06

**Authors:** Ling Lu, Ruting Xie, Rong Wei, Chunmiao Cai, Dexi Bi, Dingzi Yin, Hu Liu, Jiayi Zheng, Youhua Zhang, Feifei Song, Yaohui Gao, Linhua Tan, Qing Wei, Huanlong Qin

**Affiliations:** ^1^ Department of Pathology Shanghai Tenth People’s Hospital Affiliated to Tongji University China; ^2^ Department of Gastrointestinal Surgery Shanghai Tenth People’s Hospital Affiliated to Tongji University China; ^3^ Division of Gastroenterology Department of Medicine University of Pennsylvania Philadelphia PA USA

**Keywords:** colorectal adenocarcinoma, fibroblasts, tumor stroma, α5 integrin subunit

## Abstract

The tumorigenesis of colorectal cancer (CRC) is a complicated process, involving interactions between cancer cells and the microenvironment. The role of α5 integrin subunit in CRC remains controversial, and previous studies mainly focused on cancer cells. Herein, we report an important role of α5 in stroma fibroblasts in the tumorigenesis of CRC. The expression of α5 was found to be located in colorectal tumor stroma rather than in epithelia cancer cells. Immunofluorescence colocalization and gene correlation analysis confirmed that α5 was mainly expressed in cancer‐associated fibroblasts (CAFs). Moreover, experimental evidence showed that α5 expression was required for the tumor‐promoting effect of fibroblast cells. In an *in* *vivo* xenograft nude mice model, α5 depletion in fibroblasts dramatically suppressed fibroblast‐induced tumor growth. In an *in* *vitro* cell coculture assay, α5 depletion or knockdown reduced the ability of fibroblasts to promote cancer cell migration and invasion compared with wild‐type fibroblasts; moreover, we observed that the expression and assembly of fibronectin were downregulated after α5 depletion or knockdown in fibroblasts. Analysis of the RNA‐Seq data of the Cancer Genome Atlas cohort revealed that high expression of *ITGA5* (α5 integrin subunit) was correlated with poor overall survival in colorectal adenocarcinoma, which was further confirmed by immunohistochemistry in an independent cohort of 355 patients. Thus, our study identifies α5 integrin subunit as a novel stroma molecular marker for colorectal adenocarcinoma, offers a fresh insight into colorectal adenocarcinoma progression, and shows that α5 expression in stroma fibroblasts underlies its ability to promote the tumorigenesis of colorectal adenocarcinoma.

## Graphical abstract

The role of α5 integrin subunit in colorectal cancer (CRC) remains controversial. Here, we report an important role of α5 in stroma fibroblasts in the tumorigenesis of CRC, which might be mediated by affecting fibronectin expression and assembly. Clinically, our study showed that α5 expression was associated with overall survival in colorectal adenocarcinoma, and was positively correlated with fibronectin expression.

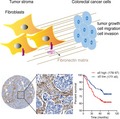

**Abbreviations**
α‐SMA: α‐smooth muscle actin
AC: classical adenocarcinoma
CAF: cancer‐associated fibroblast
CRC: colorectal cancer
ECM: extracellular matrix
FFPE: formalin‐fixed, paraffin‐embedded
GFP: green fluorescent protein
HE: hematoxylin and eosin
IHC: immunohistochemistry
MAC: mucinous adenocarcinoma
OS: overall survival
TCGA: The Cancer Genome Atlas
TGF: transforming growth factor
TMA: tissue microarray
TME: tumor microenvironment

## 1 Introduction

Colorectal cancer (CRC) is the third most commonly diagnosed cancer globally. Although 5‐year relative survival of the patients has reached almost 65% in high‐income countries, the disease remains a significant cause of mortality (Brenner *et al.*, 2014; Siegel *et al.*, 2017).

Colorectal cancer is a complex entity composed of multiple cell types and extracellular molecules in which the epithelial cancer cells actively crosstalk with the tumor microenvironment (TME). TME, or tumor stroma, which comprises fibroblasts, immune cells, blood and lymphatic vessels, and extracellular matrix (ECM), plays an important role in modulating the growth of cancer and therapeutic response through cytokines, cell–cell interactions and mechanical sensing (Bahrami *et al.*, 2018; Beauchemin, 2011). CAFs are one of the most abundant cell types in the tumor stroma and has been shown to promote tumor growth and invasion, and confer resistance to chemotherapy (Glentis *et al.*, 2017; Ishimoto *et al.*, 2017; Lotti *et al.*, 2013). Recent studies showed that CAFs could trigger cancer cell migration and invasion through assembling ECM protein‐fibronectin or intracellular signaling pathway via integrins (Attieh *et al.*, 2017; Erdogan *et al.*, 2017; Knuchel *et al.*, 2015).

Integrins are a large family of α/β heterodimeric cell adhesion molecules that mediate cell–cell, cell–ECM and cell–pathogen interactions. Integrins play an important role in signal transduction, cell migration and proliferation (Harburger and Calderwood, 2009; Hynes, 2002). The integrin subunit α5 (*ITGA5*) combines with β1 integrin subunit (*ITGB1*) to form α5β1 complex and serves as a receptor for fibronectin (Schaffner *et al.*, 2013). Ubiquitination and degradation of α5β1 integrin in lysosome are important for proper fibroblast migration on fibronectin (Lobert *et al.*, 2010), and accumulation of α5 integrin at the plasma membrane by Rab21 is required for CAFs to promote the invasion of squamous carcinoma cells *in vitro* (Hooper *et al.*, 2010). However, the significance of α5 in tumor stroma and CAFs is not well studied in CRC.

In CRC cells, the role of α5β1 is controversial, as some studies showed that α5β1 is required for colon cancer cell survival, invasion and migration (Lee and Juliano, 2000; Murillo *et al.*, 2004), whereas in another study α5β1 exerted its tumor suppressor‐like activity in colon cancer cells by inhibiting HER‐2 signaling (Kuwada *et al.*, 2005). Prognostic significance of α5β1 in CRC was identified in a study with a small cohort of patients, and α5β1 expression was shown to increase significantly in the cytoplasmic of cancer cells (Yang *et al.*, 2013). In another study, α5 integrin gene and protein levels were only increased in mucinous carcinomas but not in common colorectal adenocarcinoma (Denadai *et al.*, 2013). These conflicting reports reflect our lack of understanding of the specific role of α5 in CRC.

Herein we proposed a new model assuming that α5 expression in stroma fibroblasts was required in promoting tumor growth *in vivo* and cancer cell migration and invasion *in vitro*, accompanied by downregulated expression and assembly of fibronectin after α5 depletion in fibroblasts. Moreover, clinically we observed a positive correlation between α5 and fibronectin expression, and we showed that high expression of α5 was correlated with poor overall survival (OS) of patients with colorectal adenocarcinoma. These data suggest an important role of fibroblast‐expressed α5 in tumorigenesis of colorectal adenocarcinoma.

## 2 Materials and methods

### 2.1 Bioinformatic analysis of TCGA data

We analyzed *ITGA5* expression level using the RNA‐Seq data in 592 samples of CRC patients that with complete follow‐up information, downloaded from The Cancer Genome Atlas cohort (TCGA) database (https://portal.gdc.cancer.gov/). For the clinicopathological parameters, we included patient age, sex, tumor stage, local invasion depth (T), lymph node involvement (N), present of distant metastasis (M), lymphatic invasion and tumor location, all of which were added to the analysis of multivariate Cox regressions.

### 2.2 Hierarchical cluster analyses

Hierarchical cluster analyses were done to obtain a visual representation of gene expression correlation between *ITGA5* and CAFs markers. The hierarchical clustering algorithm used is based closely on the average‐linkage method as described previously (Eisen *et al.*, 1998) and the object of this algorithm is to compute a dendrogram that assembles all elements into a single tree. Basically, the gene expression clustering was performed using Software gene cluster 3.0, Stanford University, Califonia, USA and then visualized using Java treeview, Stanford University, Califonia, USA.

### 2.3 Patients and sample collection

Primary tumor tissues were obtained from patients with colorectal adenocarcinoma (AC) who underwent a surgical resection. A total of 355 formalin‐fixed, paraffin‐embedded (FFPE) specimens (one from each case) were collected from the Department of Pathology of Shanghai Tenth People’s Hospital Affiliated to Tongji University, between 2008 and 2013. Additionally, 24 colorectal AC tissues were collected at the time of surgical resection and were immediately snap‐frozen in liquid nitrogen before storage at −80 °C. Exclusion criteria were: (1) radiotherapy or chemotherapy before surgery; (2) presence of hereditary or inflammation‐associated CRC; (3) the presence of mucinous adenocarcinomas (MAC) or other pathological complications; (4) incomplete follow‐up information; (5) non‐CRC‐related cause of death; (6) poor quality of the tissue. According to the TNM staging system, tumors were classified independently by two pathologists. The clinicopathological parameters included were age, sex, tumor size, tumor location, pathological grade and tumor stage. All tissue samples were collected from patients with appropriate informed written consent and the study was performed in accordance with the ethical standards of the Declaration of Helsinki. The study was approved by the Ethics Committee of the hospital.

### 2.4 Tissue microarray (TMA) construction

Tissue microarray was prepared to examine the expression of α5. Briefly, a tissue core with a 0.4‐mm diameter was punched from a representative area that was examined by hematoxylin and eosin (HE) staining from FFPE tissue blocks of the primary tumor and then transferred to a recipient TMA block. Each tissue core was assigned a unique TMA location.

### 2.5 Immunohistochemistry (IHC)

Tissue microarray or whole sections of FFPE specimens were cut into 4‐μm sections and placed on polylysine‐coated slides. For immunohistochemical staining, the slides were deparaffinized in xylene and rehydrated in graded alcohols and distilled water, followed by antigen retrieval. Antigen retrieval was achieved by microwaving in 10 mm sodium citrate buffer (pH 6.0) for about 20 min. Endogenous peroxidase activity was blocked with 3% hydrogen peroxide in methanol at 37 °C for 15 min, and nonspecific protein binding was blocked with 5% goat serum. Specimens were then incubated with primary antibody against α5 (ab150361; Abcam, England or HPA002642, Sigma, St. Louis, MO, USA) overnight in a humidity chamber at 4 °C, and secondary antibody was applied for 30 min at room temperature. All sections were visualized with diaminobenzidine and counterstained with hematoxylin.

The percentage of immunostaining and the staining intensity (0, negative; 1+, weak; 2+, moderate; and 3+, strong) of the slides were recorded. A histochemistry score (*H*‐score) was calculated as described previously (Azim *et al.*, 2015) using the following formula: *H*‐score = Σ(I × Pi) = (percentage of cells of weak intensity × 1) + (percentage of cells of moderate intensity × 2) + (percentage of cells of strong intensity × 3), where *I* = intensity of staining and Pi = percentage of stained tumor cells, producing scores ranging from 0 to 300. The maximum *H*‐score would be 300, corresponding to 100% of cells with strong intensity. All sample slides were scored separately by two pathologists blinded to the clinical information.

### 2.6 Cell culture

Human colonic adenocarcinoma cell lines‐SW480 and DLD‐1 were kindly provided by Stem Cell Bank, Chinese Academy of Sciences and were maintained in Dulbecco’s modified Eagle’s medium (DMEM) (Gibco, Carlsbad, CA, USA) supplemented with 10% FBS (HyClone, Logan, UT, USA), 1% penicillin/streptomycin (P/S) (Sigma‐Aldrich, St. Louis, MO ,USA). The human normal colonic fibroblast cell line CCD‐18Co was obtained from American Type Culture Collection (ATCC, Manassas, VA, USA) and cultured according to standard protocols in ATCC‐formulated Eagle’s minimum essential medium supplemented with 10% FBS (HyClone), 1% P/S (Sigma‐Aldrich). All the cells were cultured at 37 °C supplied with 5% CO_2_. Transfection was performed with Lipofectamine 3000 (Thermo Fisher Scientific, Waltham, MA, USA).

### 2.7 *ITGA5* knock out and knock down in CCD‐18Co cell lines

*ITGA5* KO cell lines were generated using lentiCRISPR methods as described (Shalem *et al.*, 2014). Briefly, guide RNA (sgRNA) was constructed into the lentiviral expression vector with Cas9 and sgRNA (lentiCRISPR). The lentiCRISPR vector was linearized using BsmBI. The sequences of sgRNAs are: sgRNA *ITGA5*‐1#: GGGCTTCAACTTAGACGCGG; sgRNA *ITGA5*‐2#: GGGGCAACAGTTCGAGCCCA

For protein knockdown using siRNA, CCD‐18Co cells were cultured in standard conditions and transfected using HiPerFect (301704; Qiagen, Hilden, Germany). Cells were plated in 6‐well plates and subjected to transfection using 50 nm siRNA. Cells were grown for 48 h prior to use. siRNA was purchased from Qiagen, as described (Attieh *et al.*, 2017): negative control Qiagen 1027280; Integrin‐α5 oligo1 Qiagen SI00034202 CCC ATT GAA TTT GAC AGC AAA; Integrin‐α5 oligo2 Qiagen SI02654841 AAT CCT TAA TGG CTC AGA CAT.

### 2.8 Tumorigenicity assay in nude mice

All the animal studies were administered according to the guidelines of Institution Animal Care and Use Committee and all the protocols were approved by Tongji University. Male BALB/c nude mice aged 6 weeks were housed in a specific pathogen‐free environment in the Animal Laboratory Unit, Tongji University, China. SW480 cells (or DLD‐1 cells, 2 × 10^6^) with or without CCD‐18Co fibroblasts (2 × 10^6^) were suspended in 0.1 mL of PBS and injected subcutaneously into the flanks of the mice (one injection/mouse). CCD‐18Co fibroblasts alone (2 × 10^6^) were used as control. Tumor growth was monitored two times per week, and tumor volume (V) was monitored by measuring the length (L) and width (W) with a Vernier caliper and calculated using the formula V = (L × W^2^) × 0.5. After 23 or 26 days, the mice were sacrificed by cervical dislocation by an expert and qualified person, the tumor samples were collected. Tumors were harvested, FFPE and used for IHC staining.

### 2.9 Real‐time quantitative PCR (qPCR)

Total RNA was isolated from resected tissues or cultured cells using the RNeasy Mini Kit (Qiagen) according to the manufacturer’s protocol. qPCR was performed with SYBR Green Real‐time PCR Master Mix (Takara, Dalian, Japan) and quantified by the Step One Real‐Time PCR System (Applied Biosystems, Waltham, MA, USA). The sequences of primers are shown below:

*ITGA5*, forward: GCCTGTGGAGTACAAGTCCTT,

and reverse: AATTCGGGTGAAGTTATCTGTGG;

*ACTB*, forward: GTCCTGTGGCATCCACGAAACT,

and reverse: TACTTGCGCTCAGGAGGAGCAA;

*FN*, forward: CCACCCCCATAAGGCATAGG,

and reverse: GTAGGGGTCAAAGCACGAGTCATC;

*VIM*, forward: GGACCAGCTAACCAACGACA,

and reverse: AAGGTCAAGACGTGCCAGAG;

*PDGFRB*, forward: TGTGAAGGCAAGCTGGTCAA,

and reverse: ATGCGGTAACCCCGTTTGAT;

*ACTA2*, forward: CTCCGGAGCGCAAATACTCT,

and reverse: CCCGGCTTCATCGTATTCCT;

*FAP*, forward: TCCTCCAAGCAAGAAGTGTGT,

and reverse: TTCTCCAGGTACTCCTGAATCC;

*CXCL‐12*, forward: TGAGCTACAGATGCCCATGC,

and reverse: TTCTCCAGGTACTCCTGAATCC;

*IL‐6*, forward: AATGAGGAGACTTGCCTGGTG,

and reverse: TGGCATTTGTGGTTGGGTCA;

The relative expression quantitation of the target gene was determined using $2^{-\Delta \Delta C_{t}}$ method. For each sample, the assay was performed in triplicate.

### 2.10 Western blotting

The CCD‐18Co fibroblast cells were cultured until confluence and then cells starved in serum‐free medium for 48 h before myofibroblasts were differentiated by incubation of fibroblast cultures in serum‐free medium containing 10 or 20 ng·mL^‐1^ recombinant transforming growth factor (TGF)‐β1 (Peprotech, Princeton, NJ, USA) for 72 h. Subsequently, total cellular RNA was prepared as mentioned above. Cell protein was prepared as described below.

Cells were lysed using RIPA lysis buffer (Beyotime, Shanghai, China) supplemented with Halt™ Protease and Phosphatase Inhibitor Cocktail (ThermoFisher Scientific) for 30 min on ice. Supernatants were fractionated by reducing SDS/PAGE. α5 (ab150361; Abcam), β1 (ab52971; Abcam), fibronectin (ab32419; Abcam), FAPα (ab207178; Abcam), platelet‐derived growth factor receptor β (PDGFRβ) (#3169, CST), α‐smooth muscle actin (α‐SMA) (ab124964; Abcam), vimentin (ab92547; Abcam) and β‐actin (70‐ab008; MultiSciences, Hangzhou, China) were detected by immunoblotting with corresponding antibodies, and detection was performed using ECL reagent (GE Healthcare).

### 2.11 Immunofluorescence microscopy

Immunofluorescence was performed according to standard protocols (Abcam). Paraffin‐embedded sections were deparaffinized in xylene and rehydrated in graded alcohols and distilled water, followed by antigen retrieval. Antigen retrieval was achieved by microwaving in 10 mm sodium citrate buffer (pH 6.0) for about 20 min. The slides were blocked at room temperature for 60 min using blocking reagent with 5% goat serum. Specimens were then incubated with primary antibody overnight in a humidity chamber at 4 °C. These antibodies included α5 (ab150361; Abcam or MAB1969M; Merck Millipore, Burlington, MA, USA), β1 (ab52971; Abcam), α‐SMA (ab124964; Abcam), vimentin (ab92547; Abcam) and fibronectin (ab32419; Abcam). Secondary antibodies were applied for 60 min at 4 °C using Alexa Fluor 568 goat anti‐rabbit IgG or Alexa Fluor 647 goat anti‐mouse IgG (Invitrogen, Carlsbad, CA, USA). DAPI staining was included in the mounting medium. Images were obtained using a Zeiss 710 confocal microscope, Oberkochen, Germany.

SW480‐GFP and CCD‐18Co (vector or sgRNA) coculture cells or CCD‐18Co (vector or sgRNA) monoculture cells were grown on glass coverslips for 24 h and then cells were fixed with 4% formaldehyde and permeabilized with 0.1% Triton X‐100. Followed blocking for 60 min with PBS containing 1% BSA, 5% goat serum, primary antibody was incubated overnight at 4 °C with an antibody against α5 (ab150361; Abcam) or fibronectin (ab32419; Abcam). After three rinses, the cells were stained with Alexa Fluor 568 goat anti‐rabbit IgG (Invitrogen) for 1 h at room temperature and cells incubated with Phalloidin‐iFluor 647 (ab176759; Abcam). DAPI staining was included in the mounting medium. Images were obtained using a Zeiss 710 confocal microscope.

### 2.12 Cell migration and invasion assay

The Transwell migration assay was performed as described (Otomo *et al.*, 2014), SW480‐GFP cells (1 × 10^4^) alone, or together with CCD‐18Co (vector or sgRNA) cells (1 × 10^4^), were suspended in 500 μL of serum‐free DMEM, and then plated onto 8.0‐μm pore size of 24‐well Transwell® inserts (Corning, Corning, NY, USA). The bottom well was filled with 750 μL of DMEM containing 5% FBS. After being incubated at 37 °C with 5% CO_2_ for 16 h, the cells on the upper surface of the Transwell insert were scraped using cotton swabs. The migrated cancer cells on the lower surface were observed under a fluorescence microscope (Leica, Wetzlar, Germany). The same principles were used for the invasion assays; inserts contained a thin Matrigel® layer (Corning) and were plated with double‐numbered SW480‐GFP cells (2 × 10^4^) alone or together with CCD‐18Co (vector or sgRNA) cells (2 × 10^4^). Invasion was allowed to occur for 22 h.

For testing the effect of α5 knockdown in fibroblasts, DLD‐1‐GFP cells were plated together with CCD‐18Co cells treated with or without siRNA (Mock, siCtr or siα5), and the same principles were used for cell migration and invasion assay.

### 2.13 Statistical analyses

spss 22.0 (SPSS Inc., Chicago, IL, USA) and graphpad prism v.7 (La Jolla, CA, USA) were used for statistical analyses. The Kaplan–Meier method was performed to estimate OS with log‐rank test. Cox proportional hazards model was used to compare hazard ratios in both uni‐ and multivariable analyses. The multivariable analysis included known clinical relevant parameters. Results are presented in the multivariable analysis as hazard ratios including 95% confidence intervals. The correlations between *ITGA5* expression and clinicopathological features were analyzed using Chi‐square or Fisher’s exact test wherever appropriate. Pearson’s correlation was used to analyze correlations between the expression of *ITGA5* and CAFs markers. Each experiment was repeated independently a minimum of three times in the same conditions. Acquired data were presented as mean values, and error bars represent the SEM. Student’s *t*‐test was used for comparison between two groups, and a one‐way ANOVA was used to compare multiple groups with Bonferroni's multiple comparison correction. For all analyses, two‐tailed* P*‐values below 0.05 were considered significant: ^*^ *P* < 0.05, ^**^P < 0.01, ^***^P < 0.001.

## 3 Results

### 3.1 α5 Integrin subunit located in the tumor stroma and mainly expressed in CAFs

**Figure 1:**
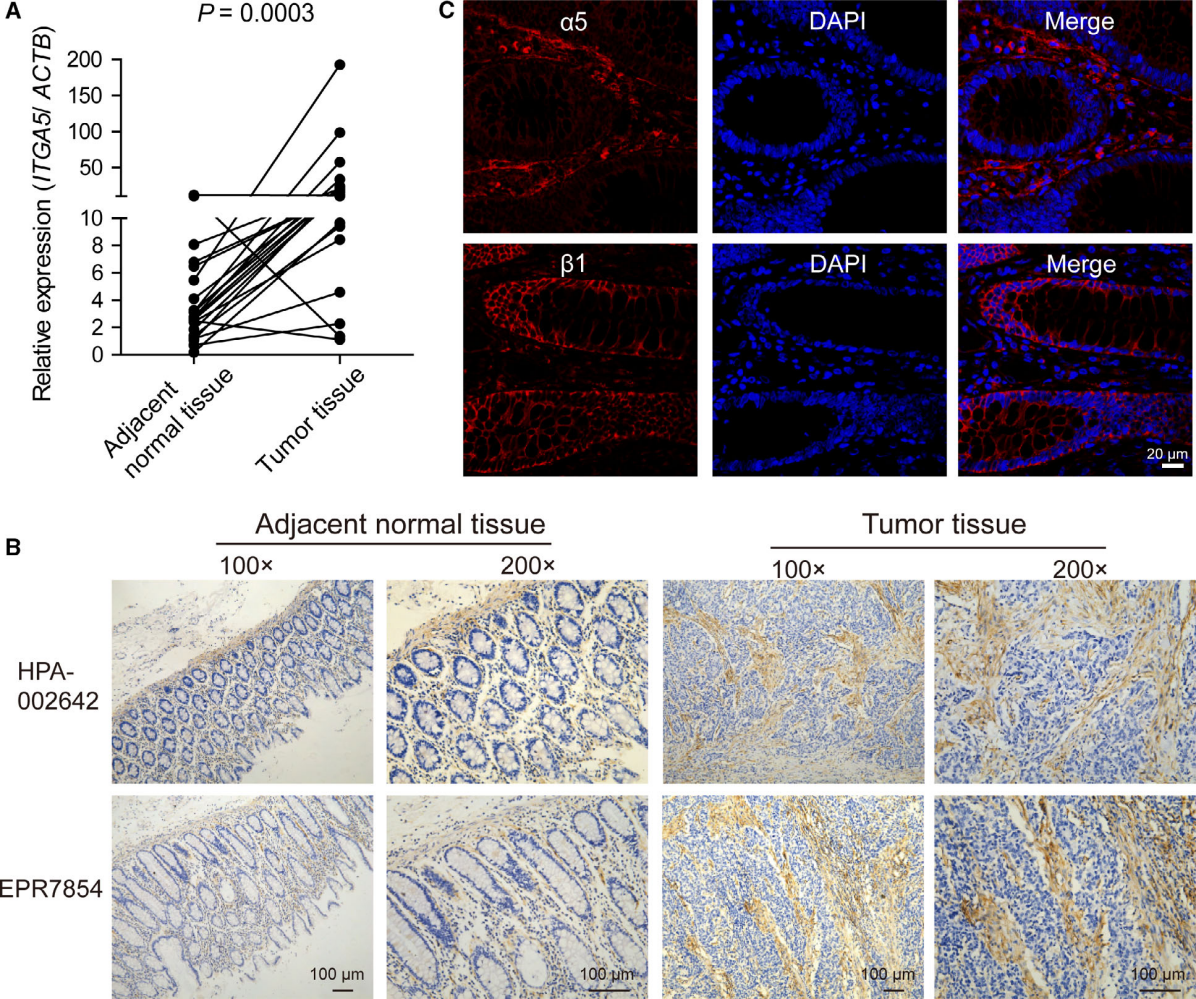
α5 Integrin expression was upregulated in colorectal adenocarcinoma and largely located in tumor stroma. (A) mRNA expression of *ITGA5* in 24 pairs of tumors and matched adjacent normal tissues. Each sample was done in triplicate, and Student’s paired *t*‐test was used for statistical analysis. (B) IHC staining of α5 in whole‐slide tissue sections of colorectal adenocarcinoma and adjacent normal tissues with two independent anti‐α5 antibodies: a polyclonal antibody (HPA‐00264) or a monoclonal antibody (EPR7854). Magnification: ×100, ×200. Scale bar: 100 μm. (C) Immunofluorescent staining for α5 integrin subunit (red) or β1 integrin subunit (red) with DAPI (blue nuclei) in human adjacent normal tissues. Scale bar: 20 μm.

To investigate the specific role of α5 integrin subunit in CRC, we first examined the expression level of α5 in freshly isolated tumor and adjacent normal tissues from 24 patients using qPCR. We observed that most tumor tissue samples from patients with colorectal adenocarcinoma (21/24; 87.5%) showed elevated levels of *ITGA5* compared with the corresponding adjacent normal tissue samples (*P* = 0.0003; Fig. 1A). We used IHC to analyze further the expression pattern of α5 integrin in tumor. Surprisingly, we found that α5 was largely expressed in the tumor stroma, whereas only weak to negligible expression levels were observed in tumor epithelial cells. In normal tissues, α5 integrin also mainly located in the lamina propria and muscularis mucosae. The result was confirmed using two completely independent antibodies against α5 in whole‐slide tissue sections of colorectal adenocarcinoma and normal adjacent tissue (Fig. 1B). Furthermore, we performed immunofluorescence staining to examine the location of α5 in human normal colorectal tissues. As shown in Fig. 1C, α5 integrin was mainly located in the lamina propria rather than epithelial cells, whereas β1 integrin subunit was largely expressed on epithelial cells as reported (Desgrosellier and Cheresh, 2010).

**Figure 2:**
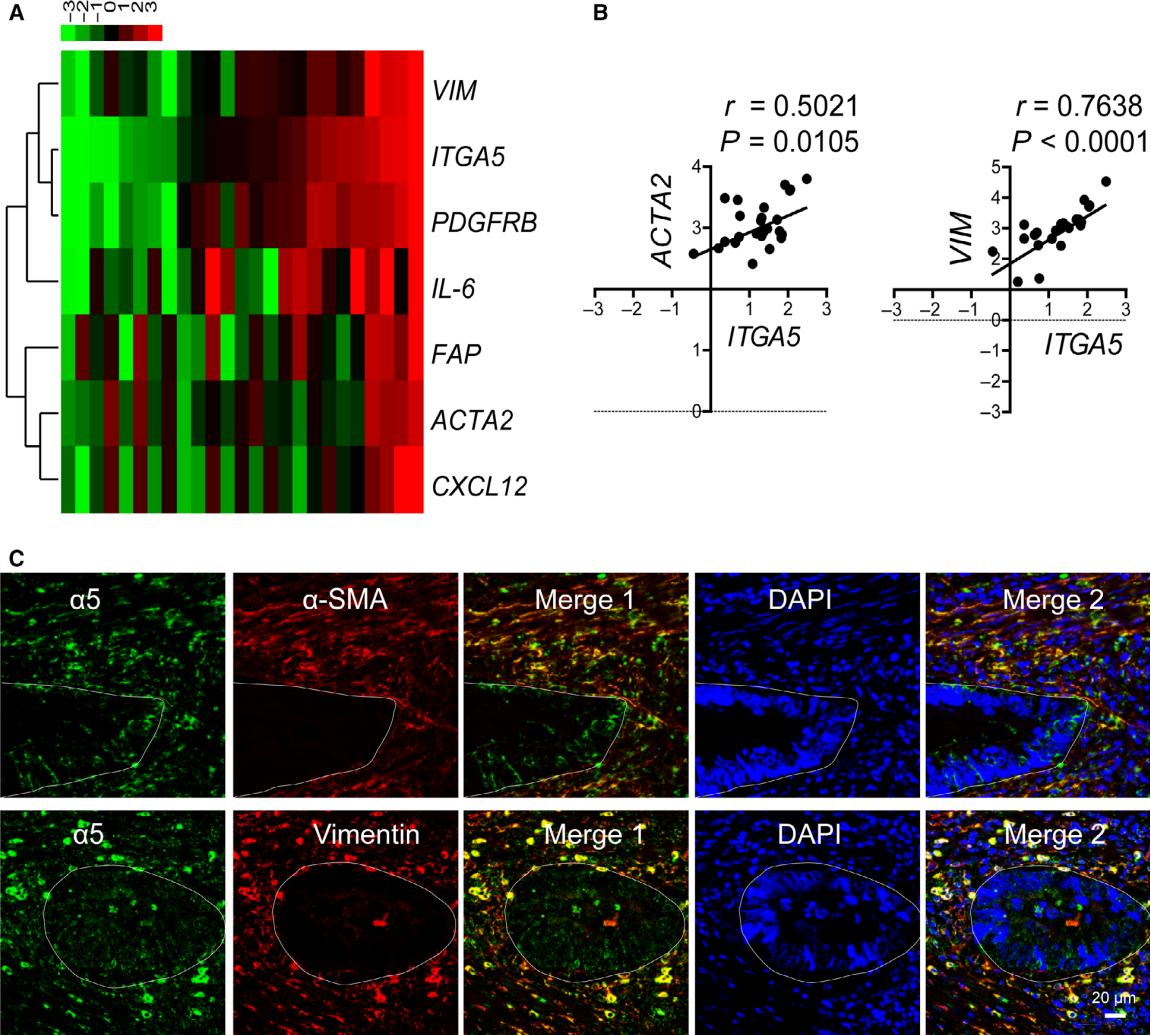
Expression of α5 was positively correlated with CAF markers in human colorectal adenocarcinomas. (A) Heatmap of the mRNA levels of *ITGA5* and CAF marker genes in 24 fresh colorectal adenocarcinoma samples evaluated by qPCR. Each row represents a gene and each column represents a case. Cases were organized from left to right by the ascending order of *ITGA5* expression level. Data were normalized to median and log_2_ values were used. The dendrogram was drawn with the hierarchical clustering method. The color scheme representing gene expression levels (row *Z*‐score) was illustrated above the heatmap. (B) Pearson’s correlation between the expressions of *ITGA5* and CAF marker genes (*ACAT2* and *VIM*) in 24 fresh colorectal adenocarcinoma samples. Expression was determined by qPCR in triplicate. (C) Representative double immunofluorescent staining of α5 (red) and CAF markers (α‐SMA or vimentin; green) with DAPI (blue nuclei) in the clinical samples with colorectal adenocarcinomas. Scale bar: 20 μm. White lines delimitate the area of tumor epithelial cells.

Carcinomas are heterogeneous tissues composed of cancer cells and stromal cells, and CAFs have been shown to be the most predominant cell type within the stromal components of carcinomas (Orimo and Weinberg, 2006). Thus we analyzed the correlation between the expression levels of *ITGA5* and CAF‐related marker genes in 24 freshly isolated colorectal adenocarcinoma samples by assessing the mRNA expression using qPCR. The CAF‐related markers are comprised mainly of six genes – *ACTA2* (α‐SMA), *VIM* (vimentin), *PDGFRB* (platelet‐derived growth factor receptor β, PDGFRβ), fibroblast activation protein (*FAP*), *CXCL‐12* (stromal cell‐derived factor 1α, SDF‐1α) and *IL‐6* (interleukin‐6) – which were selected based on previous reports (Rasanen and Vaheri, 2010). The heat map showed similar expression patterns between *ITGA5* and CAF marker genes (Fig. 2A), and calculated Pearson's correlation coefficient showed that *ITGA5* was significantly correlated with these CAF marker genes (Figs 2B and S1A). The correlation was confirmed by analyzing the RNA‐seq database of 517 colorectal adenocarcinomas extracted from TCGA cohort (Fig. S1B). These findings suggested that the expression of *ITGA5* was highly correlated with CAF marker genes and *ITGA5* might be expressed mainly in CAF cells in tumor stroma. We further confirmed the correlation by costaining FFPE colorectal adenocarcinoma samples with antibodies against α5 (green) and two CAF markers: α‐SMA (red) and vimentin (red). We noted that α5 colocalized in the tumor stroma with α‐SMA and vimentin, whereas tumor epithelial cells stained less (Fig. 2C). The pathological histology type of the sample was confirmed by HE staining (Fig. S1C). These results strongly suggested that α5 was mainly expressed in CAFs rather than epithelial cancer cells, which implied an important role of α5 in tumor fibroblasts.

### 3.2 *ITGA5* knockout in fibroblasts diminished tumor‐promoting effect of fibroblasts in nude mice

**Figure 3:**
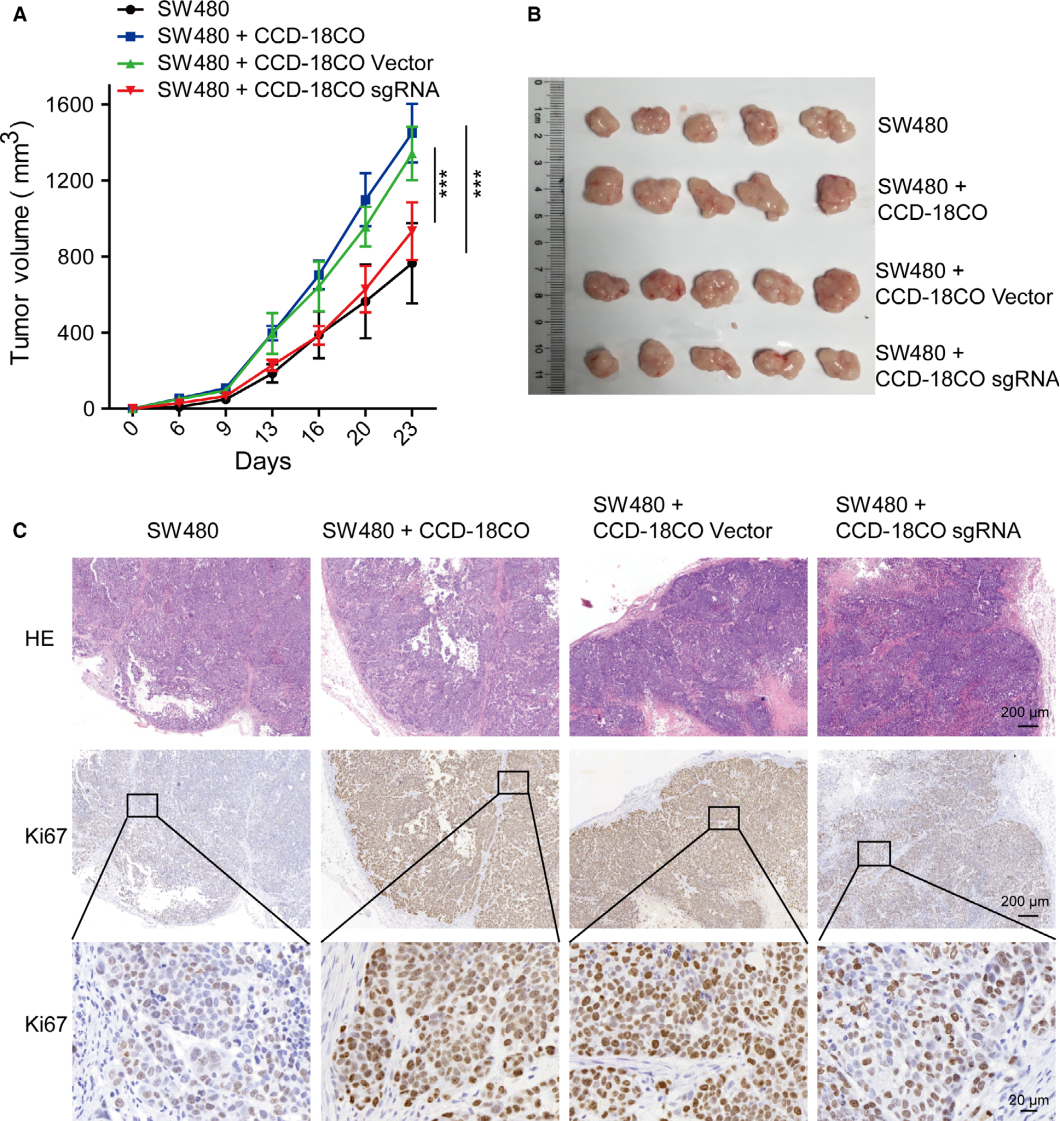
Knockout of α5 expression attenuated tumor‐promoting effect of fibroblasts. (A) The growth curves of xenograft tumors in nude mice (*n* = 5 in each group) that were injected with adenocarcinoma cancer cells (SW480) alone, SW480 and wild‐type human normal colonic fibroblasts (SW480 + CCD‐18Co), SW480 and transfected fibroblasts with α5 depletion (SW480 + CCD‐18Co sgRNA), or SW480 and transfected fibroblasts with vector control (SW480 + CCD‐18Co vector). Tumor size was measured at the indicated time points (days). Tumors were excised for histology at the last time point. Data are presented as mean ± SEM. ***P < 0.001 vs control (two‐way ANOVA with Bonferroni post‐tests). (B) Photographs of dissected tumor samples. (C) Representative HE staining and IHC staining of Ki67 in serial sections of paraffin‐embedded xenograft tumor tissues. Scale bars: 200 μm. The magnified views of the regions in black boxes are shown below; scale bar: 20 μm.

Cancer‐associated fibroblasts are a diverse cell population that can have different characteristics in different tumor types and tissue locales. A prominent component of CAFs is myofibroblasts, an activated form of fibroblasts. Fibroblasts have been widely described to have a profound influence on the development and progression of carcinoma (Noel *et al.*, 1993; Park *et al.*, 2016; Rasanen and Vaheri, 2010), as also confirmed in our *in vivo* experiments by injecting epithelial colorectal adenocarcinoma cells SW480, alone or with human normal colonic fibroblast cells CCD‐18Co, into immunocompromised (*nu/nu*) mice. The tumorigenic ability of SW480 cells was significantly promoted when co‐injected with equal numbers of CCD‐18Co cells, with significantly enhanced tumor growth and generated tumors that averaged twice the size of those formed by SW480 cells alone (Fig. S2A,B), whereas CCD‐18Co cells alone had no capacity to develop tumors (Fig. S2C). To examine whether α5 expression in fibroblasts could affect tumor progression, we compared the tumor‐promoting capacities of CCD‐18Co cells with or without α5 expression when co‐injected with epithelial colorectal adenocarcinoma cells SW480 or DLD‐1 in SCID mice. Our data showed that the tumor‐promoting effect of CCD‐18Co cells was significantly reduced after α5 was depleted by specific sgRNA (CCD‐18Co sgRNA) compared with control cells infected with empty vector (CCD‐18Co vector) (Figs 3A,B and S2D,E). Furthermore, Ki67 expression was evaluated by IHC in solid tumor tissues; a significant inhibition of Ki67 signaling was observed in solid tumors from mice co‐injected with SW480 and CCD‐18Co sgRNA cells compared with SW480 and CCD‐18Co vector cells (Fig. 3C). These data indicated that the expression of α5 integrin subunit was required for fibroblasts to promote tumor growth in nude mice.

### 3.3 α5 Depletion in fibroblasts downregulated fibronectin assembly and alleviated cancer cell migration and invasion elicited by fibroblasts

**Figure 4:**
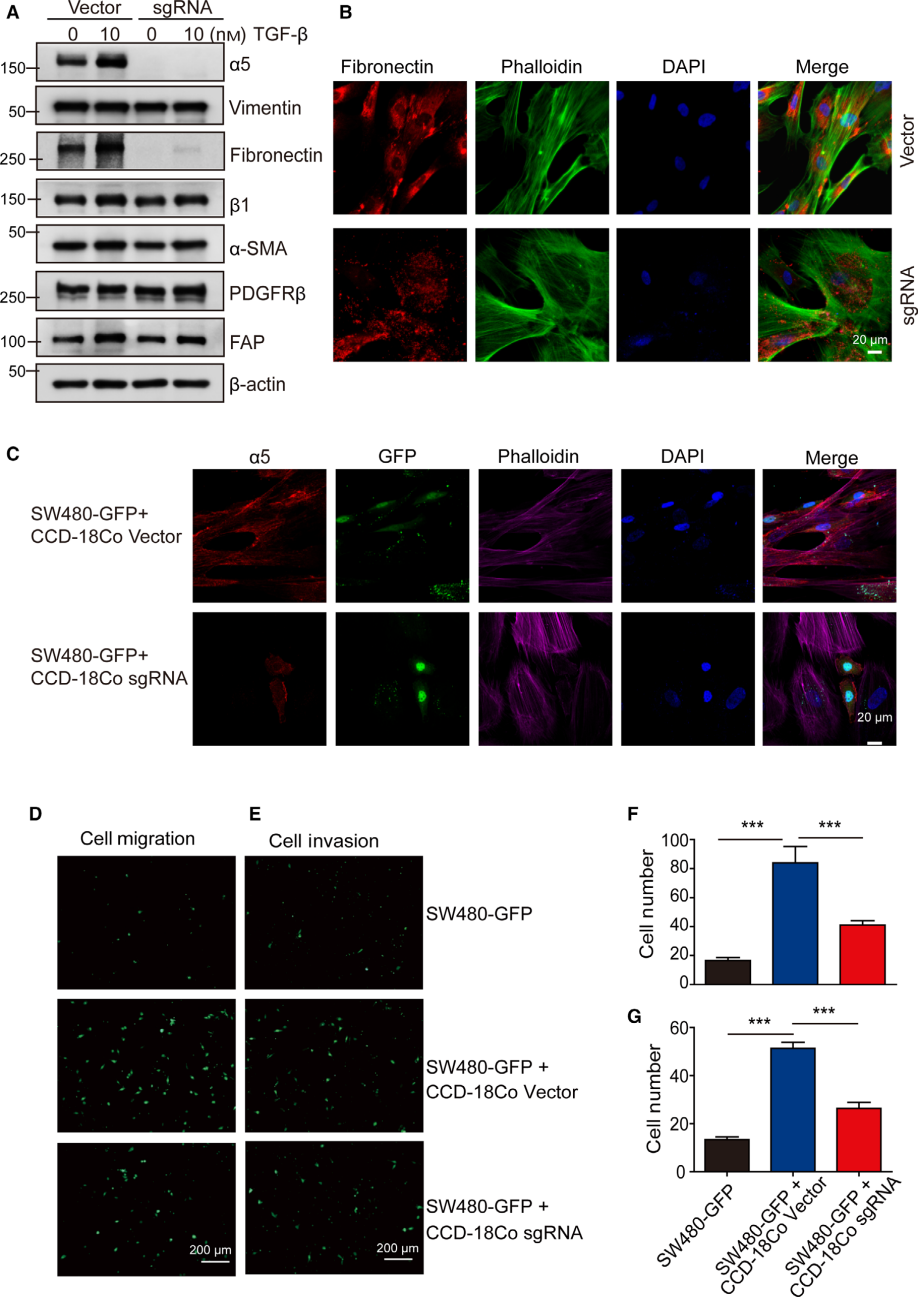
α5 Depletion downregulated fibronectin assembly in fibroblasts and showed reduced effects on promoting cancer cell migration and invasion. (A) Expression levels of α5 and selected markers were determined by western blotting in CCD‐18Co vector cells and CCD‐18Co sgRNA cells with or without TGF‐β activation. β‐Actin served as loading control. A representative result of three independent experiments is shown. (B) Immunostaining of fibronectin (red) in CCD‐18Co vector cells and CCD‐18Co sgRNA cells. F‐actin is stained with phalloidin‐647 (red) and DNA was stained with DAPI (blue). Scale bar: 20 μm. (C) α5 Depletion in fibroblasts affected cancer cell morphology in the coculture system. Representative images of SW480‐GFP cocultured with control fibroblasts (CCD‐18Co vector) or with α5‐depleted fibroblasts (CCD‐18Co sgRNA). The α5 was shown in red, F‐actin was stained with phalloidin‐647 (purple) and DNA was stained with DAPI (blue). Scale bar: 20 μm. (D–G) The α5 depletion in fibroblasts showed reduced effects on promoting cancer cell migration and invasion compared with the wild‐type fibroblasts. SW480‐GFP cells were cultured alone or cocultured with CCD‐18Co vector control cells or with α5‐depleted CCD‐18Co cells (CCD‐18Co sgRNA) in Transwell inserts with or without Matrigel. (D,F) Serum‐induced Transwell migration calculated after 16 h. SW480‐GFP cells migrating to the lower chamber were observed under a fluorescence microscope and were counted. Scale bar: 200 μm. (E,G) Serum‐induced cell invasion calculated after 22 h. SW480‐GFP cells that invaded through the Matrigel to the lower chamber were observed under a fluorescence microscope and were counted. Scale bar: 200 μm. Error bars, SEM (*n* = 3). ***P < 0.001 (one‐way ANOVA).

Preexisting normal stromal fibroblasts could potentially convert into myofibroblasts in response to TGF‐β *in vivo*, specifically during the course of tumor progression. Myofibroblasts are activated fibroblasts that are commonly characterized by the expression of multiple activation markers, including α‐SMA, FAP, PDGFRβ and vimentin (Kojima *et al.*, 2010; Midgley *et al.*, 2013). To elucidate why α5 expression was required for fibroblasts to promote tumor growth, we proceeded to examine whether α5 expression could affect fibroblast activation induced by TGF‐β. Western blotting was used to assess the protein expression of fibroblast‐activated markers in CCD‐18Co cells after TGF‐β treatment (Fig. S3A). Although CCD‐18Co cells expressed α‐SMA, FAP, PDGFRβ and vimentin in the absence of TGF‐β, the presence of gradually elevated concentrations of TGF‐β (10 and 20 ng·mL^‐1^) dramatically enhanced the expression levels of α‐SMA and FAP (Fig. S3A). Meanwhile, the expression levels of α5 and β1 subunits, especially their ligand‐fibronectin, was also progressively upregulated in the presence of elevated concentrations of TGF‐β (Fig. S3A). We further examined fibroblast activation induced by TGF‐β after α5 was depleted. Western blotting assay confirmed the depletion of α5 expression in CCD‐18Co sgRNA cells compared with vector control cells (CCD‐18Co vector cells). The depletion of α5 had no effect on the expression of β1 subunit or fibroblast‐activated markers, but the expression level of fibronectin was dramatically reduced in CCD‐18Co sgRNA cells (Fig. 4A). We confirmed the decreased expression of *FN* after α5 depletion using qPCR assay (Fig. S3B). In addition, immunofluorescence assay showed that fibronectin expression was significantly decreased in CCD‐18Co sgRNA cells, most of the fibronectin existing as spots and almost completely abrogated fibronectin fibrillogenesis, compared with CCD‐18Co vector control cells (Fig. 4B). Collectively, these observations suggested that α5 depletion on fibroblasts significantly reduced the expression of fibronectin and affected the ensuing fibronectin assembly.

Fibronectin is a major component in ECM and plays an essential role in orchestrating the assembly of ECM fibers. ECM provides a framework for cell adhesion and movement and is responsible for some morphological changes in tumor cells (Kinsey *et al.*, 2008; Mao and Schwarzbauer, 2005). Thus, we proceeded to investigate the influence of fibroblast‐expressed α5 on cancer cell morphology and function. We generated GFP‐labeled SW480 (SW480‐GFP) cells and monitored the effect of fibroblasts on CRC cell function through contact assay by coculturing SW480‐GFP cells with CCD‐18Co (vector or sgRNA) cells. We observed that SW480‐GFP cells were closely associated with fibroblasts and acquired rather elongated shapes when cultured with CCD‐18Co vector cells, whereas in the presence of CCD‐18Co sgRNA cells, SW480‐GFP cells exhibited a rounded morphology and remained discrete from fibroblasts in fluorescence microscopy (Fig. 4C). We then performed cell migration and cell invasion assay to test the role of fibroblasts on CRC cell function. The migration assay showed that a contact coculture with CCD‐18Co vector cells markedly enhanced SW480‐GFP cancer cell Transwell migration compared with that of SW480‐GFP coculturing with CCD‐18Co sgRNA cells (Fig. 4D,F). Similarly, the invasiveness of SW480‐GFP cells induced by CCD‐18Co vector cells was significantly inhibited by α5 depletion in CCD‐18Co cells (Fig. 4E,G). All of the above results implied that α5 expression on fibroblasts played an essential role in enhancing cancer cell migration and invasion, which could be mediated by affecting fibronectin expression and assembly.

Additionally, to confirm the role of α5 expression on fibroblasts *in vitro*, we tried to knock down the expression of α5 using siRNA in CCD18‐Co cells. Cells were treated with no siRNA (Mock) or siRNA targeting integrin α5 (α5 oligo1 + α5 oligo2, siα5); nontargeting siRNA was used as a negative control (siCtr). Western blotting was utilized to determine the ability of siRNA to knockdown α5 expression in CCD‐18Co cells. Cells transfected with α5 siRNA showed a reduction in α5 protein expression when compared with the mock and control siRNA‐transfected cells (Fig. S4A). Consistently the expression level of fibronectin was reduced in CCD‐18Co siα5 cells (Fig. S4A). Immunofluorescence assay showed that fibronectin fibrillogenesis was also reduced in CCD‐18Co siα5 cells compared with CCD‐18Co Mock and siCtr cells (Fig. S4B). Furthermore, we performed cell migration and cell invasion assay to test the role of fibroblasts on CRC cell function; the result showed that the transmigration and invasiveness of DLD‐1‐GFP cells were significantly decreased when cocultured with CCD‐18Co siα5 cells compared with CCD‐18Co wild‐type cells (Mock and siCtr) (Fig. S4C,E and D,F). Collectively, the above results confirmed that α5 expression on fibroblasts played an essential role in enhancing cancer cell migration and invasion, possibly mediated by their effect on fibronectin expression and assembly.

### 3.4 Clinical significance of α5 integrin expression in colorectal adenocarcinoma

**Figure 5:**
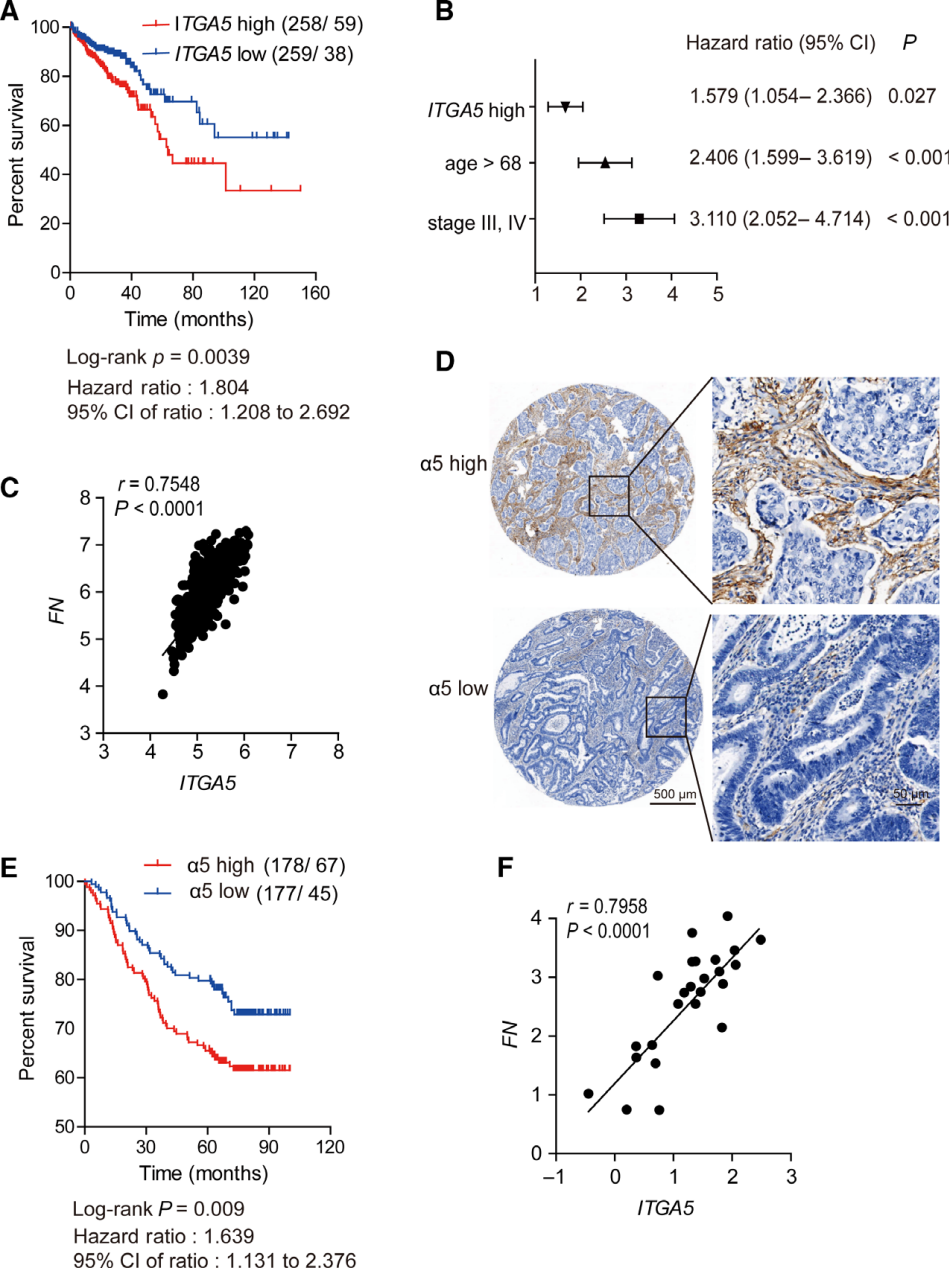
The α5 integrin expression predicted OS and was positively correlated with fibronectin expression in colorectal adenocarcinoma. (A) Kaplan–Meier curve of the OS of the patients with colorectal adenocarcinoma stratified by *ITGA5* expression in TCGA cohort. OS was analyzed with Kaplan–Meier curve and log‐rank test. (B) Cox multivariate regression shows that the stage and age of *ITGA5* expression were independent prognostic predictors (*P* < 0.05) in colorectal adenocarcinoma of TCGA cohort. Main effects are shown as hazard ratios with 95% confidence intervals. (C) Pearson’s correlation between the expressions of *ITGA5* and *FN* in 517 colorectal adenocarcinomas extracted from TCGA database. (D) Representative IHC staining (TMA section) of high or low α5 expression in an independent colorectal adenocarcinoma cohort (*n* = 355). Scale bar (left): 500 μm. Right, magnified views of the regions in the black box. Scale bar: 50 μm. (E) High expression of α5 was associated with poor prognosis in TMAs of colorectal adenocarcinoma. (F) Pearson’s correlation between the expressions of *ITGA5* and *FN* in 24 fresh colorectal adenocarcinoma samples. Expression was determined by qPCR in triplicate.

Colorectal cancer can be classified into several subtypes by histology, in which classical adenocarcinoma (AC) accounts for the large majority of cases, and about 10% of all CRC are MAC, which retained a substantial amount of mucin within the tumor. Several studies have reported on the distinct molecular and genome signatures between these different subtypes (Dunkin *et al.*, 2018; Nitsche *et al.*, 2013; Park *et al.*, 2015). In this study, to investigate the clinical significance of α5 expression in CRC, we initially utilized a bioinformatics approach and analyzed gene expression data from TCGA database. Of the 592 CRC cases that had complete follow‐up information, 517 were AC and 75 MAC. We separately stratified the AC and MAC cases into high (above the median level) and low (below the median level) expression of *ITGA5* for OS analysis. The result showed that high *ITGA5* level was associated with decreased patient survival in the AC group (HR = 1.804; 95% CI 1.208–2.692; log‐rank *P* = 0.0039) (Fig. 5A) but not in the MAC group (HR = 0.8914; 95% CI 0.3390–2.344; log‐rank *P* = 0.05436) (Fig. S5A). In addition, we assessed multivariate Cox regressions that included age, sex, tumor stage, lymphatic invasion, tumor location and *ITGA5* expression level in the colorectal AC group. The expression level of *ITGA5*, age and stage emerged as independent prognostic factors (Fig. 5B). Thus α5 integrin might act as a potential prognostic biomarker for colorectal adenocarcinoma.

Next, we analyzed the correlation between *ITGA5* expression level and clinicopathological parameters in AC groups (Table 1). We divided all the cases into two groups according to the expression status of *ITGA5*. *ITGA5* expression level was observed to be significantly associated with local invasion depth (*P* = 0.002) and lymph node involvement (*P* = 0.041) (Table 1). We also observed a significantly correlated expression between *ITGA5* and *FN* (*P* < 0.0001; *r* = 0.7548, Pearson’s correlation), as shown in Fig. 5C.

**Table 1:** Characteristics of 517 colorectal adenocarcinoma cases according to *ITGA5* expression levels in TCGA cohort.

Characteristics^a^	All cases,	*ITGA5* expression levels		*P*
	*n *= 517	High expression, *n =* 258 (49.9%)	Low expression, *n* *= *259 (50.1%)	
Age				
> 68	247 (47.8%)	118 (45.7%)	129 (49.8%)	0.354^b^
≤ 68	270 (52.2%)	140 (54.3%)	130 (50.2%)	
Sex				
Men	279 (54.0%)	140 (54.3%)	139 (53.7.8%)	0.892^b^
Women	238 (46.0%)	118 (45.7%)	120 (46.3%)	
Tumor location				
Proximal colon	210 (40.6%)	100 (38.8%)	110 (42.5%)	0.644^b^
Distal colon	211 (40.8%)	107 (41.5%)	104 (40.2%)	
Rectum	96 (18.6%)	51 (19.8%)	45 (17.4%)	
Local invasion depth				
T1/T2	115 (22.2%)	43 (16.7%)	72 (27.8%)	0.002^b^
T3/T4	402 (77.8%)	215 (83.3%)	187 (72.2%)	
Lymph node involvement				
N0	302 (58.4%)	138 (53.5%)	164 (63.3%)	0.041^b^
N1	122 (23.6%)	64 (24.8%)	58 (22.4%)	
N2	93 (18.0%)	56 (21.7%)	37 (14.3%)	
Distant metastasis				
M0	444 (85.9%)	217 (84.1%)	227 (87.6%)	0.258^c^
M1	73 (14.1%)	41 (15.9%)	32 (12.4%)	
Tumor stage				
0/I/II	300 (58.0%)	140 (54.3%)	160 (61.8%)	0.084^b^
III/IV	217 (42.0%)	118 (45.7%)	99 (38.2%)	
Lymphatic invasion				
Yes	193 (37.3%)	105 (40.7%)	88 (34.0%)	0.114^b^
No	324 (62.7%)	153 (59.3%)	171 (66.0%)	

^a^ Percentages in all cases or in the corresponding subgroups (high expression or low expression) are given in the brackets. ^b^χ^2^ test. ^c^Fisher’s exact test.

To confirm further the clinical significance of α5 expression in Chinese patients with AC, we selected 355 eligible patients diagnosed with colorectal AC in 2008–2013 and assessed integrin α5 expression by IHC staining of the TMA block. The cases were divided into two groups according to the expression status of α5 (high or low) (Fig. 5D). Of the 355 cases, 112 (31.5%) patients died during a median follow up of 68.23 months (from 0.13 to 100.23 months). The association between α5 expression levels and OS of the patients was analyzed using Kaplan–Meier analysis and log‐rank test. A statistically significant difference in OS was observed between these two groups of patients; the group with high expression of α5 had a worse prognosis than the other group (HR = 1.639; 95% CI 1.131–2.376; log‐rank *P* = 0.009) (Fig. 5E). The clinicopathological parameters of this independent group were summarized according to the staining status of α5 in Table S1. Furthermore, univariate Cox regression model analysis revealed that older age (*P* = 0.013), advanced TNM stage (*P* < 0.001) and high expression of α5 (*P* = 0.010) were associated with poor prognosis of CRC in terms of OS (Table 2). When these three factors were included into the multivariate Cox regression model, the results showed that older age (HR = 1.753; 95% CI 1.200–2.561; *P* = 0.004), advanced TNM stage (HR = 2.000; 95% CI 1.372–2.916; *P* < 0.001) and high expression of α5 (HR = 1.621; 95% CI 1.110–2.366; *P* = 0.012) were independent prognostic factors for colorectal adenocarcinoma (Table 2). Consistently, *ITGA5* was significantly correlated with *FN* (*P* < 0.0001; *r* = 0.7958, Pearson’s correlation) in 24 freshly isolated colorectal adenocarcinoma samples by assessing the mRNA expression using qPCR (Fig. 5F). We also examined the colocalization between α5 (green) and fibronectin (red), which showed positive colocalization (Fig. S5B). Thus, α5 expression was clinically correlated with fibronectin expression and α5 expression in tumor stroma might serve as a potential prognostic marker in colorectal adenocarcinoma.

**Table 2:** Univariate and multivariate Cox regression analyses for the independent cohort with 355 colorectal adenocarcinoma cases. CI, confidence interval; HR, hazard ratio.n.

Characteristics	Univariate analysis			Multivariate analysis^a^		
	HR	95% CI	*P*	HR	95% CI	*P*
Age (> 63 vs ≤ 63)	1.612	1.106–2.350	0.013	1.753	1.200–2.561	0.004
Sex (Male vs female)	0.915	0.631–1.326	0.637			
Tumor size (> 4 vs ≤ 4)	1.167	0.806–1.691	0.414			
Tumor location			0.721			
Distal colon vs proximal colon	0.845	0.525–1.359	0.487			
Rectum vs proximal colon	1.02	0.666–1.563	0.928			
Tumor stage (III/IV vs 0/I/II)	1.935	1.331–2.815	< 0.001	2.000	1.372–2.916	< 0.001
Grade (low vs medium or high)	0.888	0.477–1.655	0.710			
α5 (high vs low expression)	1.646	1.128–2.402	0.010	1.621	1.110–2.366	0.012

^a^ Age, tumor stage and α5 expression levels were included for multivariate analysis.

## 4 Discussion

Our efforts uncovered an important role of α5 in a tumor‐supporting effect of fibroblasts and suggested that α5 might act as a new stroma molecular signature for colorectal adenocarcinoma. The expression of α5 was shown to be largely expressed in tumor fibroblasts and was required for fibroblasts in promoting tumor growth *in vivo* and supporting cancer cell migration and invasion *in vitro*. Clinically, high α5 expression level was associated with poor OS in colorectal adenocarcinoma, and correlation analysis showed that the expression level of α5 was significantly associated with local tumor invasion depth and lymph node metastasis (Table 1), which further suggested the tumor‐promoting role of α5 in fibroblasts. Thus stroma expression of α5 might be a therapeutic target for CRC patients with adenocarcinoma. Interestingly, Kuninty *et al*. (2018) recently reported that α5 integrin could also contribute to tumor‐promoting effects of pancreatic stellate cells in pancreatic cancer, and revealed α5 as a therapeutic target in pancreatic cancer, which suggested a ubiquitous role of α5 expressed on fibroblasts to promote tumorigenesis.

To identify the specific cells in the tumor stroma that mainly expressed α5, we performed gene correlation analysis and co‐immunostaining assay between *ITGA5* (α5 integrin subunit) and CAF markers, as CAFs have been shown to be abundantly present within the stromal components of carcinomas (Orimo and Weinberg, 2006). The results showed an extraordinarily correlated expression between α5 and CAF markers (Figs 2 and S1), suggesting that stroma expression of α5 mainly occurred in CAFs. Myofibroblasts are a prominent component of CAFs and have been suggested to produce pro‐invasive signals that induce a more aggressive phenotype in the tumor (Liang *et al.*, 2005; Nakayama *et al.*, 1998). We showed that depletion of α5 greatly reduced the tumor‐promoting effect of fibroblasts, implying an important role of α5 in CRC through tumor fibroblasts. Our work was done using a normal colonic fibroblast cell line, which supports our final conclusion, as there are reports showed that preexisting normal stromal fibroblasts could potentially convert into myofibroblasts in response to TGF‐β *in vivo*, specifically during the course of tumor progression (Kojima *et al.*, 2010; Midgley *et al.*, 2013). We understand that primary isolated CAF might support these conclusions better, and therefore more experiments will be performed to further this study using primary CAFs in the future.

In our experiments, the expression level of α5 and fibronectin was significantly increased together with fibroblast activation markers during the process of fibroblast‐myofibroblasts conversion induced by TGF‐β (Figs 4A and S3A). However, α5 depletion in fibroblasts did not affect the expression levels of fibroblast activation markers as shown in the western blotting assay (Fig. 4A), except for significant inhibition of fibronectin expression and assembly (Figs 4A,B and S3B). In a recent report, TGF‐β was shown to induce peritumoral fibronectin deposition and tumor infiltration in basal cell carcinoma (Kuonen *et al.*, 2018), which suggested that α5 might affect the expression and assembly of fibronectin through some TGF‐β responsive pathways, rather than a general effect on the activation state of fibroblasts. Our following study showed that cancer cell migration and invasion induced by fibroblasts was significantly inhibited by α5 depletion in *in vitro* coculture assays, which might due to the downregulated assembly of fibronectin. These results were consistent with two recent reports, as fibronectin assembly has been shown to be a new hallmark of CAFs, and the ability of fibroblasts to induce cancer cell invasion was directly correlated with the amount of fibronectin they assembled (Attieh *et al.*, 2017). Also, alignment of fibronectin fibers by CAFs could promote CAF‐cancer cell interactions and mediate directional cancer cell migration in coculture assays, whereas knocked down fibronectin expression in CAFs completely disrupted ECM synthesis and organization (Erdogan *et al.*, 2017).

A proangiogenic role of α5β1 has been well demonstrated (Kim *et al.*, 2000; Muether *et al.*, 2007; Schaffner *et al.*, 2013; Taverna and Hynes, 2001) and has been implicated in a phase I clinical study that used dual anti‐angiogenic therapy combining α5β1 and vascular endothelial growth factor inhibition in patients with advanced solid tumors (Weekes *et al.*, 2018). Although no overt clinical activity was observed in that study (Weekes *et al.*, 2018) the authors found that the highest expression of fibronectin in hepatocellular carcinoma (HCC) patients showed the best overall response. Evidence has accumulated showing a crucial role of fibronectin assembly in supporting tumor progression (Wang and Hielscher, 2017; Yoneda, 2015); however, the relationship between the expression of fibronectin and the prominent role of integrin α5β1 in tumor stroma has not been well elucidated. Fibronectin is assembled into fibers through its binding to integrin adhesion receptors. Integrin α5β1 is the major fibronectin receptor and has been shown to be responsible for fibronectin matrix assembly (Kinsey *et al.*, 2008). Consistent with previous studies identifying a crucial role of α5β1 in mediating fibronectin matrix formation in CAFs (Erdogan *et al.*, 2017; Hooper *et al.*, 2010), we found that α5 expression in CCD‐18Co fibroblasts indad a great effect on fibronectin assembly (Figs 4B and S4B) by reducing the expression of fibronectin, as confirmed by western blotting assay (Figs 4A and S4A). All of these data reinforced the interactions between α5 and fibronectin. Although we preliminarily found that the downstream PI‐3‐kinase signaling pathway was significantly influenced after α5 was depleted (data not shown), it remains unclear how α5 depletion affects fibronectin expression and thus more experiments are needed to study the underlying mechanism further.

## 5 Conclusions

In summary, we demonstrate in the current study that α5 integrin subunit was mainly located in the colorectal tumor fibroblasts, and we provide evidence that α5 expression is required for fibroblasts to exhibit a tumor‐promoting effect, as α5 depletion in fibroblasts dramatically suppresses fibroblast‐induced tumor growth in xenograft nude mice and inhibits cancer cell migration and invasion induced by fibroblasts in *in vitro* coculture assays. We also observed that the expression and assembly of fibronectin were downregulated after α5 was depleted in fibroblasts. Our study offered fresh insight into colorectal adenocarcinoma progression and shows that α5 expression on tumor stroma might serve as a prognostic marker and therapeutic target in colorectal adenocarcinoma.

## Conflict of interest

The authors declare no conflict of interest.

## Author contributions

LL, QW and HLQ designed the research study; LL and RTX performed the majority of the experiments and data analysis; RW and LHT helped with experiments; CMC assisted with *in vivo* experiments and part of the research design; DXB helped to download and analyze the TCGA data; JYZ and HL contributed to specimen preparation; YHZ assisted with confocal microscopy; DZY, FFS and YHG helped with interpreting results. The manuscript was drafted by LL and edited by HLQ and QW. All authors approved the final version of the manuscript.

## Supporting information


**Fig. S1.** (A) Pearson's correlation between the expressions of *ITGA5 *and CAF marker genes (*PDGFRB*, *FAP*, *IL‐6 *and *CXCL‐12*) in 24 fresh colorectal adenocarcinoma samples. Expression was determined by qPCR in triplicate. (B) Heat map of mRNA transcript levels of CAF marker genes in 517 colorectal adenocarcinomas extracted from TCGA database. Each row represents a gene and each column represents a case. Cases were organized from left to right by the ascending order of *ITGA5 *expression level. The dendrogram was drawn with the hierarchical clustering method. The color scheme representing gene expression levels (row Z‐score) is illustrated above the heatmap. (C) Representative HE staining of the samples used for double immunofluorescent staining in Fig. 3C. Scale bar: 50 μm.
**Fig. S2.** (A,B) Tumor growth stimulated by fibroblasts. Nude mice (*n* = 7 in each group) were injected with adenocarcinoma cancer cells alone (SW480) or with human normal colonic fibroblasts (SW480+ CCD‐18Co). (A) The growth curves of the xenograft tumors. Tumor size was measured at the indicated time points (days). Data are presented as mean ± SEM. *** *P *< 0.001 vs control (two‐way ANOVA with Bonferroni post‐tests). Tumors were excised for histology at the last time point. (B) Photographs of dissected tumor samples. (C) Photographs of nude mice that were injected with human normal colonic fibroblasts (CCD‐18Co) alone after 25 days. (D) The growth curves of xenograft tumors in nude mice (*n *= 7 in each group) that were injected with adenocarcinoma cancer cells (DLD‐1) alone, DLD‐1 and transfected fibroblasts with α5 depletion (SW480+ CCD‐18Co sgRNA) or DLD‐1 and transfected fibroblasts with vector control (SW480+ CCD‐18Co vector). Tumor size was measured at the indicated time points (days). Tumors were excised for histology at the last time point. Data are presented as mean ± SEM. *** *P *< 0.001 vs control (two‐way ANOVA with Bonferroni post‐tests). (E) Photographs of dissected tumor samples.
**Fig. S3.** (A) Expression levels of α5 and selected markers were determined by western blotting in CCD‐18Co cells with or without TGF‐β activation. A representative result of three independent experiments is shown on the left. Quantification of three independent blots for α5 expression as a ratio relative to the expression level of control cells is shown on the right. Data are presented as mean ± SEM. *** *P *< 0.001 vs control (one‐way ANOVA). (B) Transcript levels of *ITGA5 *and *FN *determined by qPCR. Experiments were performed in triplicate and Student’s *t*‐test was used for statistical analysis.
**Fig. S4.** (A) Expression levels of α5 and fibronectin were determined by western blotting in CCD‐18Co cells treated with no siRNA (Mock) or siRNAs (α5 oligo1+ α5oligo2) targeting integrin α5 (siα5); non‐targeting siRNA was used as a negative control (siCtr). β‐Actin served as loading control. A representative result of three independent experiments is shown. (B) Immunostaining of fibronectin (red) in CCD‐18Co cells treated with or without siRNA. F‐actin is stained with phalloidin‐647 (red) and DNA is stained with DAPI (blue). Scale bar: 20 μm. (C–F) The α5 knockdown in fibroblasts shows reduced effects on promoting cancer cell migration and invasion compared with the wild‐type fibroblasts. DLD‐1‐GFP cells were co‐cultured with wild‐type CCD‐18Co cells (Mock and siCtr) or with α5 knockdown CCD‐18Co cells (siα5) in Transwell inserts with or without Matrigel. (C,E) Serum‐induced Transwell migration was calculated after 16 h. DLD‐1‐GFP cells migrating to the lower chamber were observed under a fluorescence microscope and were counted. Scale bar: 200 μm. (D,F) Serum‐induced cell invasion were calculated after 22 h. DLD‐1‐GFP cells that invaded through the Matrigel to the lower chamber were observed under a fluorescence microscope and were counted. Scale bar: 200 μm. Error bars, SEM (*n *= 3). * *P *< 0.05 (one‐way ANOVA).
**Fig. S5.** (A) Kaplan–Meier curve of the overall survival of the patients with MAC stratified by *ITGA5 *expression in TCGA cohort. Overall survival was analyzed with Kaplan–Meier curve and log‐rank test. (B) Representative double immunofluorescent staining of α5 (red) and fibronectin (green) with DAPI (blue nuclei) in the clinical samples with colorectal adenocarcinomas. Scale bar: 20 μm. White lines delimitate the area of tumor epithelial cells.
**Table S1.** Characteristics of 355 CRC cases according to α5 staining status in the independent cohort.
