# Supplementary material for: Integrin α5 subunit is required for the tumor supportive role of fibroblasts in colorectal adenocarcinoma and serves as a potential stroma prognostic marker
Source: Mol Oncol. 2019 Nov 6;13(12):2697–714. doi: 10.1002/1878-0261.12583 (PMC6887586; doi:10.1002/1878-0261.12583)
Supplement: Supplementary file 1 — Fig. S1. (A) Pearson's correlation between the expressions of ITGA5 and CAF marker genes (PDGFRB, FAP, IL‐6 and CXCL‐12) in 24 fresh colorectal adenocarcinoma samples. Expression was determined by qPCR in triplicate. (B) Heat map of mRNA transcript levels of CAF marker genes in 517 colorectal adenocarcinomas extracted from TCGA database. Each row represents a gene and each column represents a case. Cases were organized from left to right by the ascending order of ITGA5 expression level. The dendrogram was drawn with the hierarchical clustering method. The color scheme representing gene expression levels (row Z‐score) is illustrated above the heatmap. (C) Representative HE staining of the samples used for double immunofluorescent staining in Fig. 3C. Scale bar: 50 μm. Fig. S2. (A,B) Tumor growth stimulated by fibroblasts. Nude mice (n = 7 in each group) were injected with adenocarcinoma cancer cells alone (SW480) or with human normal colonic fibroblasts (SW480+ CCD‐18Co). (A) The growth curves of the xenograft tumors. Tumor size was measured at the indicated time points (days). Data are presented as mean ± SEM. *** P < 0.001 vs control (two‐way ANOVA with Bonferroni post‐tests). Tumors were excised for histology at the last time point. (B) Photographs of dissected tumor samples. (C) Photographs of nude mice that were injected with human normal colonic fibroblasts (CCD‐18Co) alone after 25 days. (D) The growth curves of xenograft tumors in nude mice (n = 7 in each group) that were injected with adenocarcinoma cancer cells (DLD‐1) alone, DLD‐1 and transfected fibroblasts with α5 depletion (SW480+ CCD‐18Co sgRNA) or DLD‐1 and transfected fibroblasts with vector control (SW480+ CCD‐18Co vector). Tumor size was measured at the indicated time points (days). Tumors were excised for histology at the last time point. Data are presented as mean ± SEM. *** P < 0.001 vs control (two‐way ANOVA with Bonferroni post‐tests). (E) Photographs of dissected tumor samples. Fig. S3. (A) E [file MOL2-13-2697-s001.pdf]

Supplementary Figures

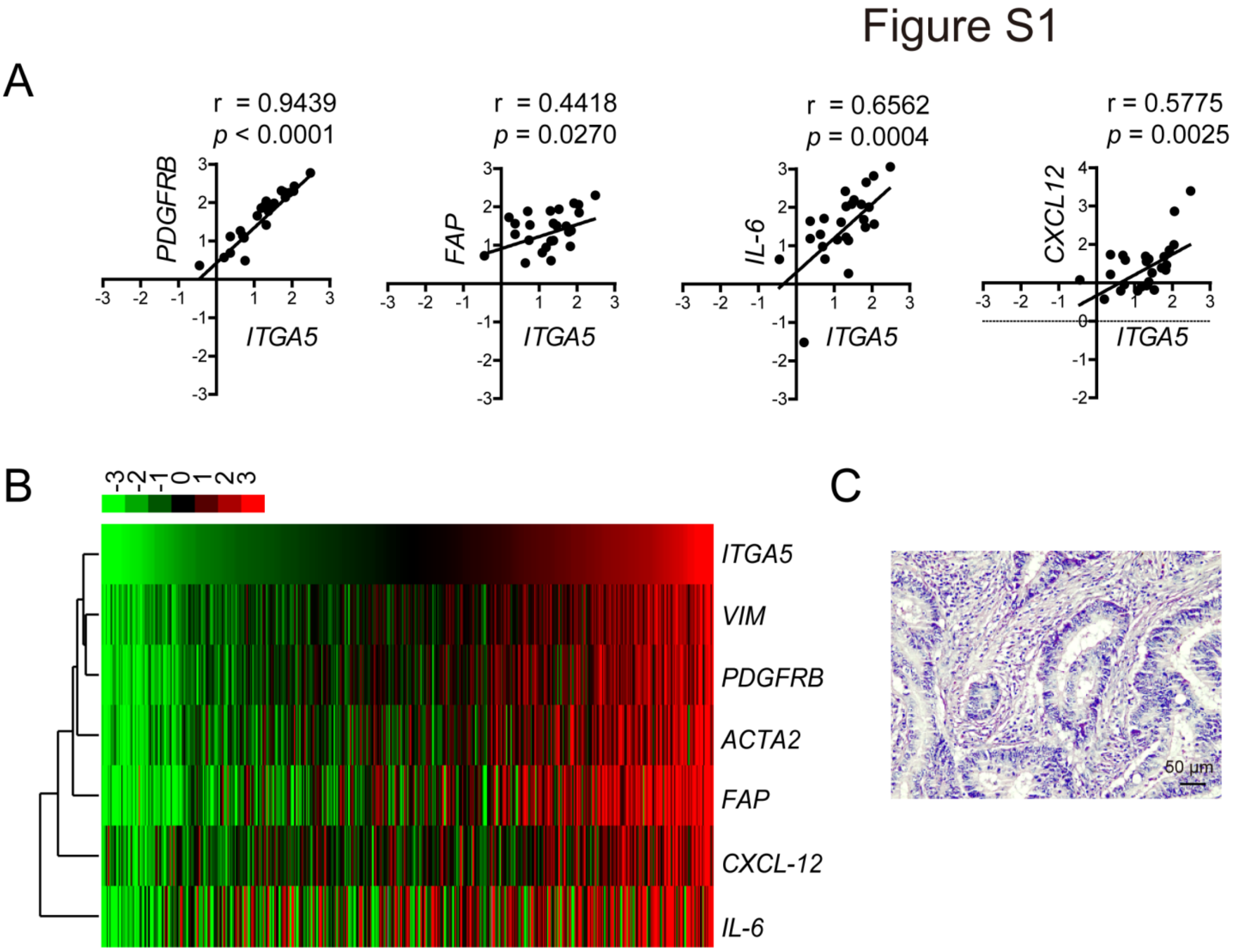

Figure S1. (A) Pearson's correlation between the expressions of *ITGA5* and CAF marker genes (*PDGFRB*, *FAP*, *IL-6* and *CXCL-12*) in 24 fresh colorectal adenocarcinoma samples. Expression was determined by qPCR in triplicate. (B) Heat map of mRNA transcript levels of CAF marker genes in 517 colorectal adenocarcinomas extracted from TCGA database. Each row represents a gene and each column represents a case. Cases were organized from left to right by the ascending order of *ITGA5* expression level. The dendrogram was drawn with the hierarchical clustering method. The color scheme representing gene expression levels (row Z-score) was illustrated above the heatmap. (C) Representative HE staining of the samples used for double immunofluorescent staining in Fig. 3C. Scale bar, 50 μm.

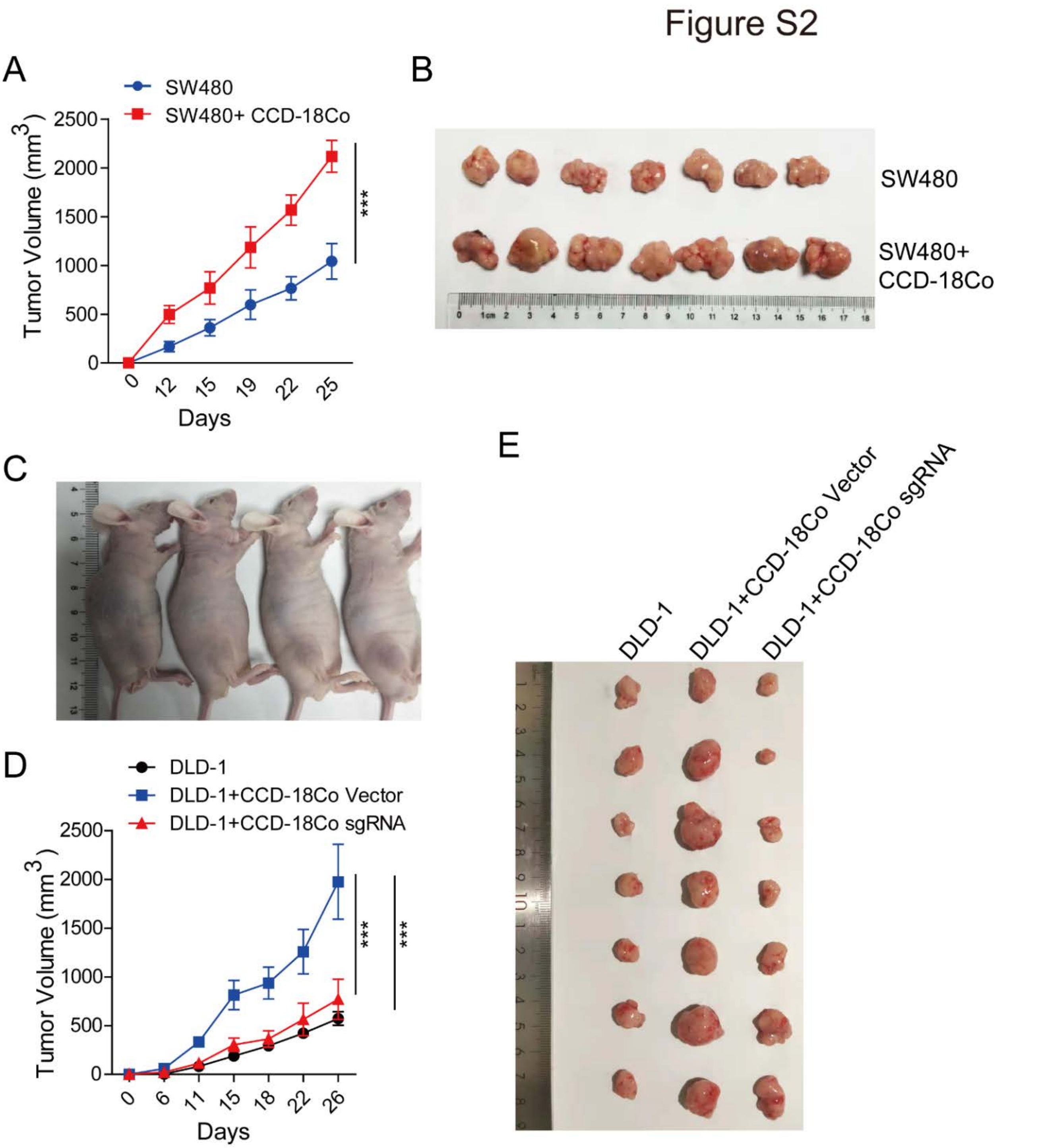

Figure S2. (A, B) Tumor growth stimulated by fibroblasts. Nude mice (n = 7 in each group) were injected with adenocarcinoma cancer cells alone (SW480) or together with human normal colonic fibroblasts (SW480+ CCD-18Co). (A) The growth curves of the xenograft tumors. Tumor size was measured at the indicated time points (days). Data are presented as mean ± s.e.m.. ***, $p < 0.001$ vs control (two-way ANOVA with Bonferroni post-tests). Tumors were excised for histology at the last time point. (B) Photographs of dissected tumor samples. (C) Photographs of nude mice that were injected with human normal colonic fibroblasts (CCD-18Co) alone after 25 days. (D) The growth curves of xenograft tumors in nude mice (n= 7 in each group) that were injected with

adenocarcinoma cancer cells (DLD-1) alone, DLD-1 and transfected fibroblasts with α5 depletion (SW480+ CCD-18Co sgRNA), or DLD-1 and transfected fibroblasts with vector control (SW480+ CCD-18Co vector). Tumor size was measured at the indicated time points (days). Tumors were excised for histology at the last time point. Data are presented as mean ± s.e.m.. ***, $p < 0.001$ vs control (two-way ANOVA with Bonferroni post-tests). (E) Photographs of dissected tumor samples.

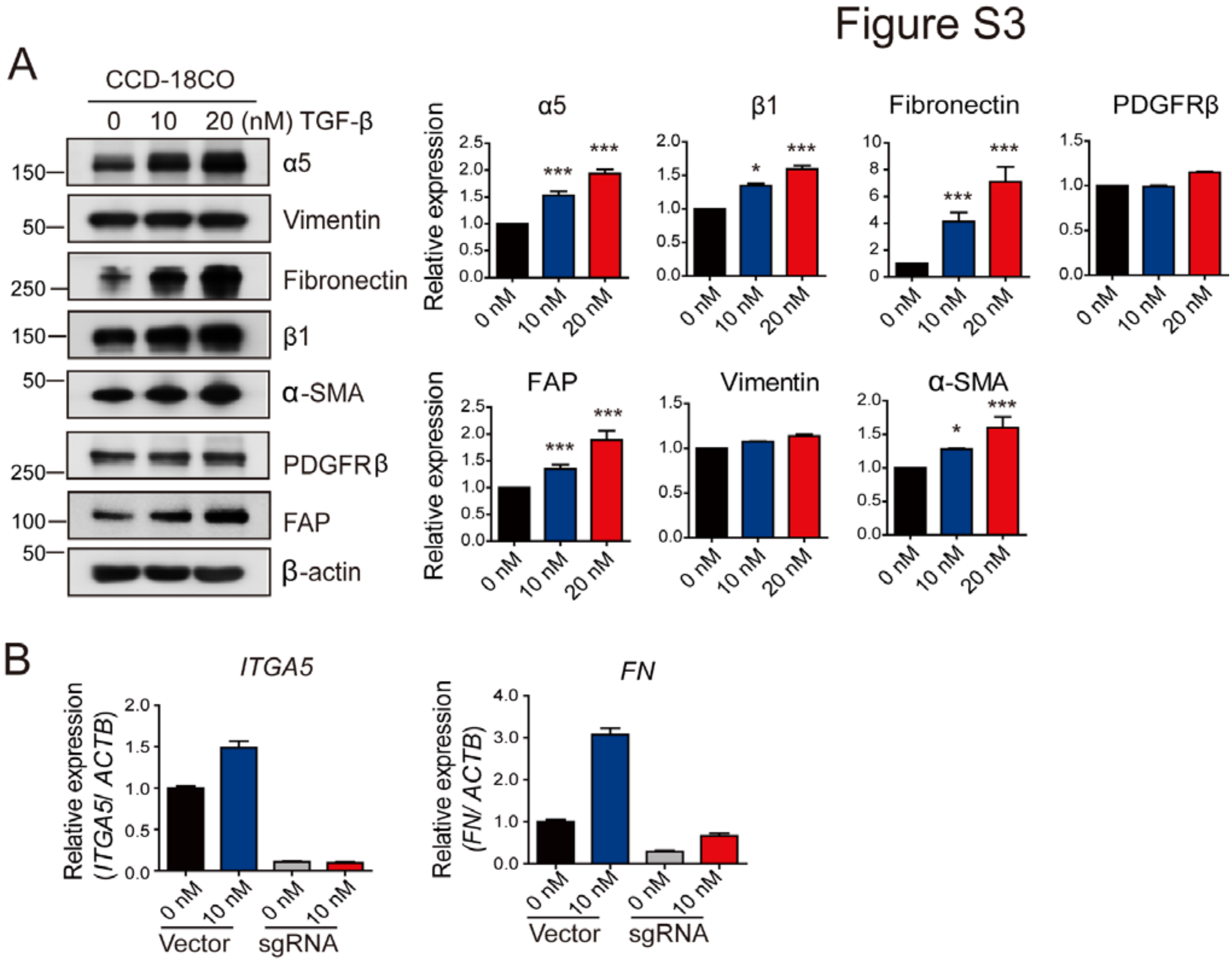

Figure S3. (A) Expression levels of α5 and selected markers were determined by western blotting in CCD-18Co cells with or without TGFβ activation. A representative result of three independent experiments is shown on the left. Quantification of three independent blots for α5 expression as a ratio relative to the expression level of control cells is shown on the right. Data are presented as mean ± s.e.m.. ***, $p < 0.001$ vs control (one-way ANOVA). (B) Transcript levels of *ITGA5* and *FN* determined by qPCR. Experiments were performed in triplicate and student's *t*-test was used for statistical analysis.

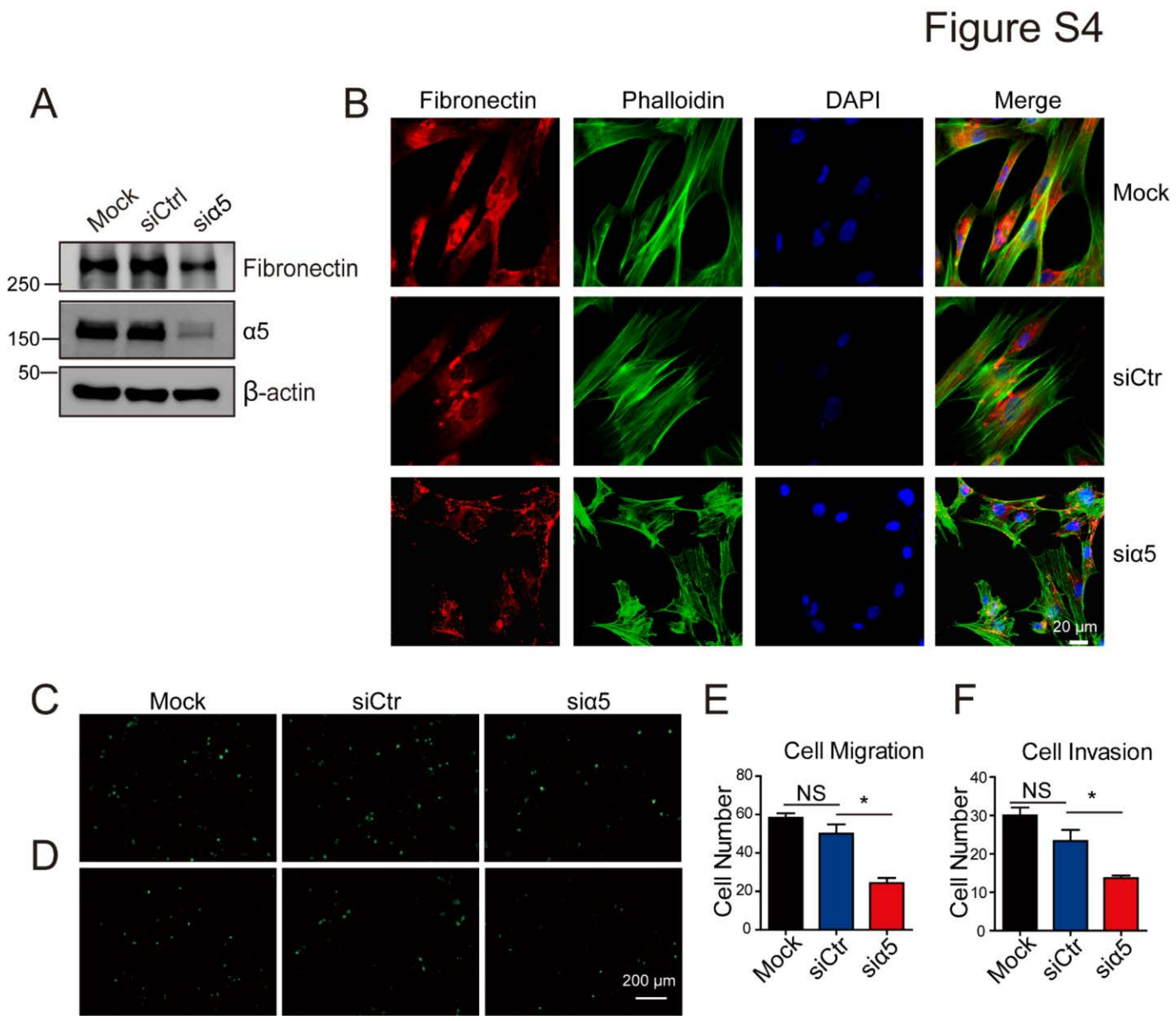

Figure S4. (A) Expression levels of α5 and fibronectin were determined by western blotting in CCD-18Co cells treated with no siRNA (Mock) or siRNAs (α5 oligo1+ α5oligo2) targeting integrin α5 (siα5), non-targeting siRNA was used as a negative control (siCtr). β-actin served as loading control. A representative result of three independent experiments is shown. (B) Immunostaining of fibronectin (red) in CCD-18Co cells treated with or without siRNA. F-actin is stained with phalloidin-647 (red), and DNA was stained with DAPI (blue). Scale bar, 20 μm. (C, D, E, F,) The α5 knockdown in fibroblasts showed reduced effects on promoting cancer cell migration and invasion compared to the wild-type fibroblasts. DLD-1-GFP cells were co-cultured with wildtype CCD-18Co cells (Mock and siCtr) or with α5 knockdown CCD-18Co cells (siα5) in transwell inserts with or without matrigel. After 16 hours, (C, E) Serum-induced transwell migration were calculated. DLD-1-GFP cells migrating to the lower chamber were observed under a fluorescence microscope and were counted. Scale bar, 200 μm. After 22 hours, (D, F) Serum-induced cell invasion were calculated. DLD-1-GFP cells that invaded through the matrigel to the lower chamber were observed under a fluorescence microscope and were counted. Scale bar, 200 μm. Error bars, s.e.m. (n = 3). *, $p < 0.05$ (one-way ANOVA).

Figure S5

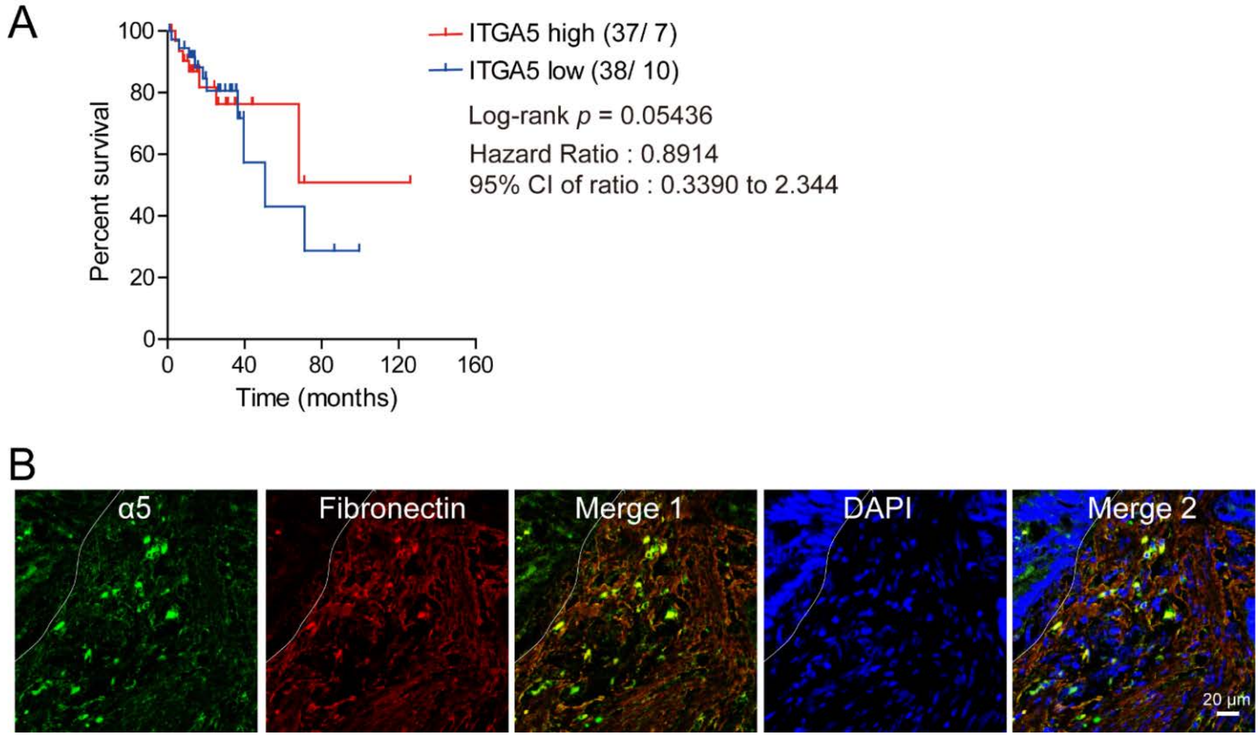

Figure S5. (A) Kaplan-Meier curve of the overall survival of the patients with MAC stratified by *ITGA5* expression in TCGA cohort. Overall survival was analyzed with Kaplan-Meier curve and Log-rank test. (B) Representative double immunofluorescent staining of α5 (red) and fibronectin (green) with DAPI (blue nuclei) in the clinical samples with colorectal adenocarcinomas. Scale bar, 20 μm. White lines delimitate the area of tumor epithelial cells.

Supplementary Table S1. Characteristics of 355 CRC cases according to α5 staining status in the independent cohort.

Characteristics[a]	All cases	α5 staining status		p
	355	High expression 177 (49.9%)	Low expression 178 (50.1%)	
Age				0.915[b]
> 63	171 (49.7%)	85(50.0%)	86 (49.4%)	
<=63	173 (50.3%)	85(50.0%)	88 (50.6%)	
Sex				0.061[b]
Men	195 (54.9%)	106 (59.9%)	89 (50.0%)	
Women	160 (45.1%)	71 (40.1%)	89 (50.0%)	
Tumor size				0.283[b]
>4 cm	160 (45.7%)	85 (48.6%)	75 (42.9%)	
<=4 cm	190 (54.3%)	90 (51.4%)	100 (57.1%)	
Tumor location				0.732[b]
Proximal colon	96 (27.0%)	46 (26.0%)	50 (28.1%)	
Distal colon	118 (33.2%)	57 (32.2%)	61 (34.3%)	
Rectum	141 (39.7%)	74 (41.8%)	67 (37.6%)	
Local invasion depth				0.468[b]
Tis/T1/T2	82(23.1%)	38 (21.5%)	44 (24.7%)	
T3/T4	273 (76.9%)	139 (78.5%)	134 (75.3%)	
Lymph node involvement				0.508[b]
N0	207 (58.3%)	98 (55.4%)	109 (61.2%)	
N1	99 (27.9%)	52 (29.4%)	47 (26.4%)	
N2	49 (13.8%）	27 (15.3%)	22 (12.4%)	
Distant metastasis				0.857[c]
M0	322 (90.7%)	160 (90.4%)	162 (91.0%)	
M1	33 (9.3%)	17 (9.6%)	16 (9.0%)	
Tumor stage				0.099[b]
0/I/II	198 (55.8%)	91 (51.4%)	107 (60.1%)	
III/IV	157 (44.2%)	86 (48.6%)	71 (39.9%)	
Grade				0.174[c]
Low	38 (10.7%)	23 (13.0%)	15 (8.4%)	
Medium or High	317 (89.3%)	154 (87.0%)	163 (91.6%)	

[a]Percentages in all cases or in the corresponding subgroups (High expression or Low expression) were given in the brackets.
[b]χ^2 test.
[c]Fisher's exact test.
